# Complete Genome Sequence of *Chryseobacterium camelliae* Dolsongi-HT1, a Green Tea Isolate with Keratinolytic Activity

**DOI:** 10.1128/genomeA.01421-17

**Published:** 2018-01-11

**Authors:** Eun-Mi Kim, Kyeong Hwan Hwang, Jun-Seong Park

**Affiliations:** a R & D Center, Amore-Pacific Corporation, Yong-In, Kyounggi-do, Republic of Korea

## Abstract

The complete genome sequence of *Chryseobacterium camelliae* Dolsongi-HT1 is reported here. *C. camelliae* Dolsongi-HT1, having keratinolytic activity, was isolated from green tea leaves in the Dolsongi tea garden in Jeju, South Korea. The strain Dolsongi-HT1 has 28 candidate protease genes, which may be utilized in further studies and industrial applications of keratinase.

## GENOME ANNOUNCEMENT

Keratin is a structural protein found in animal hair, bird feathers, nail, wool, and the epidermis of the skin (1). Keratins are highly resistant to degradation by common proteases, such as trypsin, papain, and pepsin, due to the presence of numerous disulfide bonds and structural rigidity. Some species belonging to the genus *Chryseobacterium* have been reported for their strong keratinolytic activity (2–4). However, so far, little has been reported about the characterization of keratinase or proteases, and only one complete genome sequence of a *Chryseobacterium* species strain associated with keratin degradation has been presented (5).

*Chryseobacterium camelliae* Dolsongi-HT1 was isolated in an experiment aimed to isolate keratinolytic activity from green tea leaves (*Camellia sinensis*) on M9 minimal medium containing 0.5% (wt/vol) keratin as a nitrogen source. The isolated strain was deposited in the Korea Culture Center of Microorganisms (KCCM11883P).

DNA extraction was done from overnight culture using the FastDNA SPIN kit for soil (MP Biomedicals, CA, USA). The complete genome of the *Chryseobacterium camelliae* Dolsongi-HT1 strain was constructed *de novo* using Pacific Biosciences (PacBio) sequencing data. Sequencing analysis was performed at ChunLab, Inc. (South Korea) and the National Instrumentation Center for Environmental Management (NICEM). PacBio sequencing data were assembled with SPAdes 3.10.1 (Algorithmic Biology Lab, St. Petersburg Academic University of the Russian Academy of Science) and PacBio SMRT Analysis 2.3.0 using the HGAP2 protocol (Pacific Biosciences, USA). The resulting contigs were circularized using Circlator 1.4.0 (Sanger Institute).

The length of the complete circular chromosome is 4,376,354 bp, and the average G+C content is 41.81%. No plasmids were detected. The gene-finding and functional annotation of whole-genome assembles were performed with the EzBioCloud genome database (ChunLab, Inc.). The protein-coding sequence (CDS) predicted by Prodigal 2.6.2 (6) contained 4,012 genes. Furthermore, 12 rRNA genes were identified by the Rfam 12.0 database (7), and 62 tRNA genes were identified by tRNAscan-SE1.3.1 (8).

Among the CDSs, a total of 28 open reading frames were identified as protease genes, such as those for metalloprotease (EC 3.4.21.-), serine protease (EC 3.4.21.-), zinc protease (EC 3.4.99.-), carboxyl-terminal protease, and ATP-dependent Clp protease. In addition, among the 28 candidate protease genes, 4 proteases have a signal peptide, which can be secreted into the extracellular region. The complete genome sequence of *C. camelliae* Dolsongi-HT1 not only provides insight into genetic information for keratin degradation but also shows potential application in traditional industries, such as those for detergents, leather, and cosmetics (9, 10).

### Accession number(s).

The complete genome sequence of *Chryseobacterium camelliae* Dolsongi-HT1 was deposited at GenBank under the accession number CP022986. The strain is available from the Korea Culture Center of Microorganisms (KCCM) under the accession number KCCM11883P.
